# Long Non-coding RNAs RN7SK and GAS5 Regulate Macrophage Polarization and Innate Immune Responses

**DOI:** 10.3389/fimmu.2020.604981

**Published:** 2020-12-09

**Authors:** Imran Ahmad, Araceli Valverde, Raza Ali Naqvi, Afsar R. Naqvi

**Affiliations:** Mucosal Immunology Lab, College of Dentistry, University of Illinois at Chicago, Chicago, IL, United States

**Keywords:** long noncoding RNA, macrophage polarization, phagocytosis, antigen uptake and processing, epigenetic regulation and gene expression

## Abstract

Macrophages (M*φ*) are immune cells that exhibit remarkable functional plasticity. Identification of novel endogenous factors that can regulate plasticity and innate immune functions of M*φ* will unravel new strategies to curb immune-related diseases. Long non-coding RNAs (lncRNAs) are a class of endogenous, non-protein coding, regulatory RNAs that are increasingly being associated with various cellular functions and diseases. Despite their ubiquity and abundance, lncRNA-mediated epigenetic regulation of M*φ* polarization and innate immune functions is poorly studied. This study elucidates the regulatory role of lncRNAs in monocyte to M*φ* differentiation, M1/M2 dichotomy and innate immune responses. Expression profiling of eighty-eight lncRNAs in monocytes and *in vitro* differentiated M2 M*φ* identified seventeen differentially expressed lncRNAs. Based on fold-change and significance, we selected four differentially expressed lncRNAs *viz.*, RN7SK, GAS5, IPW, and ZFAS1 to evaluate their functional impact. LncRNA knockdown was performed on day 3 M2 M*φ* and the impact on polarization was assessed on day 7 by surface marker analysis. Knockdown of RN7SK and GAS5 showed downregulation of M2 surface markers (CD163, CD206, or Dectin) and concomitant increase in M1 markers (MHC II or CD23). RN7SK or GAS5 knockdown showed no significant impact on CD163, CD206, or CD23 transcripts. M1/M2 markers were not impacted by IPW or ZFAS1 knockdown. Functional regulation of antigen uptake/processing and phagocytosis, two central innate immune pathways, by candidate lncRNA was assessed in M1/M2 M*φ*. Compared to scramble, enhanced antigen uptake and processing were observed in both M1/M2 M*φ* transfected with siRNA targeting GAS5 and RN7SK but not IPW and ZFAS1. In addition, knockdown of RN7SK significantly augmented uptake of labelled *E. coli in vitro* by M1/M2 M*φ*, while no significant difference was in GAS5 silencing cells. Together, our results highlight the instrumental role of lncRNA (RN7SK and GAS5)-mediated epigenetic regulation of macrophage differentiation, polarization, and innate immune functions.

## Introduction

The crux of immune protection lies in the conglomerate activities of innate and adaptive immune systems that eventually converge towards clearance of the pathogenic microorganisms. Macrophages (M*φ*), dendritic cells (DCs) and neutrophils are the key sentinels of human innate immune system (1–4). Leveraging a specific repertoire of receptors (*viz* toll-like receptors; TLRs), M*φ* recognize and uptake pathogens and clear them primarily by phagocytosis, in addition they also coordinate with the adaptive immune system (*e.g.*, T and B cells) by processing and presenting pathogen specific antigens to these cells (5, 6). Numerous reports have now suggested that M*φ* polarization during the recognition of patterns at the surface of a diversified pathogen *via* PPR regulates the sequence of events leading to the activation of the adaptive arm of the immune system. Binding of pathogens to immune cell receptors activates a plethora of downstream signals including chromatin-modifying complexes and transcription factors, which eventually decide the type of macrophage polarization, M1 or M2 (7–9). M1 macrophages possess a pro-inflammatory phenotype, while M2 are of anti-inflammatory (reparative) in nature. Human studies have shown that M1 and M2 polarization can be reversible upon environmental changes (10). Thus, we hypothesize that the identification of specific biomolecules leading to the development of M1 and M2 M*φ* will be helpful to discern, 1) activation status of M*φ*, 2) and resolution of the infection/inflammation.

Lately, 200 nt long, non-protein coding RNAs, dubbed as lncRNAs, are expressed in specific cell types. LncRNAs have now been classified as long intergenic ncRNAs (lincRNAs), natural antisense transcripts (NATs), transcripts of uncertain coding potential (TUCP), enhancer RNAs (eRNAs), and pseudogene-derived lncRNAs (11, 12). With the exception of lincRNAs and NATs, lncRNAs express at a lower level as compared to protein-coding genes (12). Various reports have now elucidated the role of lncRNAs in a multitude of biological processes, including the development of cardiomyocytes (13), stem cells (14), epithelial cells (15), erythrocytes (16), and adipocytes (17). Furthermore, the studies have deciphered the roles of lncRNAs in the maturation of specific immune cells *via* Hematopoietic stem cells (HSC) (18). Two lncRNAs, HOTAIRM1 and Morrbid (Myeloid RNA regulator of Bim-induced death), regulate the crucial genes involved in myeloid cell differentiation (HOXA1 and HOXA2, as well CD11b and CD18) and regulate the lifespan of short-lived myeloid cells (*e.g.* neutrophils, eosinophils, and classical monocytes), respectively (19–21).

A study by Wang et al. has unequivocally highlighted the expression bias of an lncRNA (lnc-DC) in the human conventional DC population, as compared to monocytes. This study has further divulged that the expression of lnc-DC (driven by PU.1) is instrumental in maintaining the activation status of classical DCs *via* regulating STAT3 phosphorylation pathway (22). Other studies also emphasized the role of lncRNAs in human M*φ* innate immune functions. A single exon, 793 bp long lncRNA PACER (p50-associated COX2 extragenic RNA) was found to regulate the transcription of PTGS2 (COX2) by acting as a decoy molecule in the NF-*κ*B signaling pathway (23). PACER is reported as LPS inducible lncRNA, which interacts and sequesters the repressor subunit of NF-*κ*B and therefore, enables the binding of NF-*κ*B complex binding at the promoter of PTGS2. Also, Ptgs2 divergent (Ptgs2 opposite strand; Ptgs2os) lncRNA was reported to be induced in mice fibroblast upon TLR activation (24). These studies, in a concerted manner, suggest that the expression dynamics of lncRNAs act as imperative sensors and regulate the sequence of events, eventually leading to clearance of pathogens *via* modifying the epigenetics of their targeting genetic loci.

Contemplating it, we examined the changes in lncRNA expression during M2 macrophage differentiation and addressed whether their differential expression regulates the polarization and innate immune functions (antigen uptake/processing and phagocytosis). Based on differential expression and further knockdown studies, our results divulges the key roles of lncRNAs, RN7SK, and GAS5 in regulating macrophage plasticity (M1/M2), antigen uptake/processing and phagocytosis.

## Materials and Methods

### Primary Human Monocyte Isolation and Differentiation

Peripheral blood mononuclear cells (PBMCs) were purified from freshly prepared buffy coats collected from healthy donors (n = 4; Sylvan N. Goldman Oklahoma Blood Institute, Oklahoma City, OK) using Ficoll Paque (GE Healthcare, Piscataway, NJ) based density centrifugation, as described earlier (25–30). CD14^+^ monocytes were isolated from PBMCs by incubating with magnetic-labeled CD14 beads (Miltenyi Biotec, Cologne, Germany), according to the manufacturer’s instructions. The purity of CD14^+^ cells was >95%, as determined by flow cytometry. For generation of M1 M*φ* and M2 M*φ*, monocytes were plated in DMEM (without FBS) supplemented with penicillin (100 U/ml) and streptomycin (100 mg/ml). After 2 h, media was removed and replaced with complete DMEM containing 10% FBS (Life Technologies, Grand Island, NY), and 1,000 U/ml recombinant human (rh) GM-CSF for generation of M1 or 50 ng/ml M-CSF (PeproTech, Rocky Hill, NJ) for generation of M2 macrophages. Fresh media was replaced every 72 h. At day 7, cells were harvested, and surface expression of CD14, CD163, and HLA-DR was examined by flow cytometry analysis.

### Transient siRNA Transfection

LncRNA or control siRNAs were purchased from Sigma (Germantown, MD). Transient transfection of siRNA was performed in M1 and M2 macrophages using Lipofectamine 2000 (Life Technologies), as per manufacturer’s instructions. siRNAs were used at final concentration of 100 nM. As a transfection positive control, siGLO Red Transfection Indicator is a fluorescent oligonucleotide duplex labeled with DY-547 (ThermoFisher).

### Cell Viability Assay

Cell viability was determined using the CellTiter 96 AQueous Cell Proliferation Assay Kit (Promega, Madison, WI). Briefly, M1 and M2 macrophages were differentiated in 96-well plates, transfected with lncRNA or control siRNA (100 nM), and assays were performed 24 and 48 h post transfection, according to manufacturer’s protocol.

### LncRNA Profiling Using PCR Array

Total RNA was isolated from M2 macrophages and monocytes using the miRNeasy mini Kit (Qiagen), according to the manufacturer’s instructions. First-strand cDNA was synthesized from 1 µg total RNA using the Reverse Transcription Kit (Qiagen). For lncRNA expression profiling, Human lncFinder RT² lncRNA PCR Array plate (LAHS-001Z) containing eighty-eight different lncRNAs and controls in 96-well format was used (Qiagen) using real-time PCR (StepOne Plus Thermocycler; Applied Biosystems, Carlsbad, CA). Expression levels were normalized with respect to SNORA73A, and the fold change was calculated using delta-delta CT method.

### Quantitative Real-Time Reverse Transcriptase-Polymerase Chain Reaction

Total RNA was isolated from 18 h, day 3, day 5, and day 7 differentiated cells using miRNeasy micro kit (Qiagen). A total of 250 ng RNA was used to synthesize cDNA, which was synthesized from first-strand cDNA synthesis kit (Invitrogen). The expression levels of GAS5, IPW, and *β*-actin genes were analyzed in a StepOne 7500 thermocycler (Applied Biosystems). The Ct values of three replicates were analyzed to calculate fold change using the 2^−ΔΔCt^ method.

### Flow Cytometry

Cells were harvested and washed in ice-cold PBS supplemented with 1% (v/v) FBS and 0.08% sodium azide. Cellular debris were excluded based on size (forward scatter [FSC]) and granularity (side scatter [SSC]). The FSC/SSC gate for Monocyte comprised ∼60%, total events. Couplets were excluded based on SSC *versus* FSC and SSC *versus* pulse width measurements. Samples were stained for cell surface markers with FITC, PE, and APC conjugated antibodies. For polarization analysis, human antibodies for CD23, MHCII (both M1 marker), CD163, CD206, and dectin (M2 marker) were purchased from BD Pharmingen or BioLegend. Unstained and isotype control treated cells were used as controls. Samples were analyzed using a FACScan or BD Cyan flow cytometer using CellQuest software (BD Biosciences, San Jose, CA. Further analysis was performed using FlowJo software (Tree Star, Ashland, OR). Cells were gated according to their forward scatter (FSC) and side scatter (SSC) properties including the larger cells with high granularity and excluding the small-sized debris with a low SSC and FSC shown at the bottom left corner of the dot plot.

### Antigen Uptake and Processing Assay

LncRNA or control siRNA transfected M*Φ* were challenged with a combination of Texas Red- and DQ™-conjugated Ova (1 mg/ml, Molecular Probes, Grand Island, NY) in complete media for 2–4 h at 37°C, as described earlier (26). DQ™-Ova consist of Ova that are heavily conjugated with BODIPY FL, resulting in self-quenching. Upon proteolytic degradation of DQ-Ova to single dye-labeled peptides, bright green fluorescence is observed. For flow cytometry, cells were harvested with Accutase (Cell Biolabs, San Diego, CA) treatment, washed twice with 1× PBS/0.1% BSA and analyzed on BD Fortessa (BD Biosciences, Franklin Lakes, NJ).

### Phagocytosis Assay

Macrophages (400,000/well, 96-well plate) were transfected on day 6 with lncRNA or control siRNA. Phagocytosis assay was performed with pHrodo Green conjugated *E. coli* (Invitrogen, Carlsbad, CA) 24 h post transfection, according to the manufacturer’s protocol. Briefly, the labeled *E. coli* bioparticles were resuspended in Live Imaging Buffer (Life Technologies) and homogenized by sonication for 2 min and resuspended in culture media, as described earlier (25). The cells were incubated with labeled *E. coli* for 2 h, then washed three times with PBS, fixed with 4% paraformaldehyde, and analyzed by flow cytometry.

### Statistical Analysis

All the data were analyzed and plotted using GraphPad Prism (La Jolla, USA). The results are represented as SD or SEM from three independent replicates. P-values were calculated using Students t-test, and *P <0.05* was considered significant. *P < 0.05, **P < 0.01, ***P < 0.001.

## Results

### Differential Expression of Long Non-Coding RNAs in Monocyte-Differentiated Macrophages

**Figure 1:**
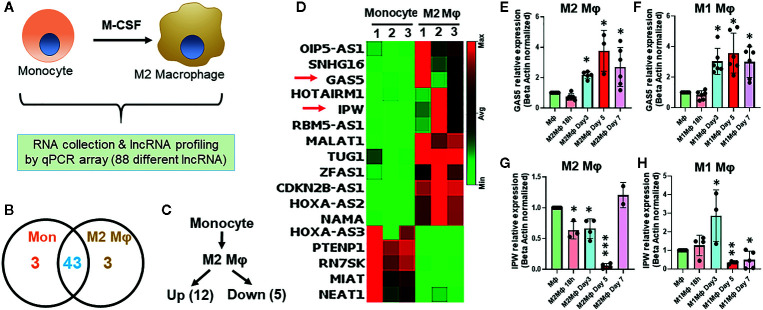
Differential expression of lncRNAs occurs in monocyte-derived macrophages. **(A)** Schematics of the experimental design to evaluate lncRNA profiles. **(B)** Venn diagram summarizing differentially expressed lncRNAs genes in monocyte and day 7 differentiated M2 macrophages. Corresponding numbers in left and right elucidate the lncRNA identified in either monocytes or M2 M*φ*. The number depicted in the overlapping region shows the number of lncRNAs expressed in monocytes and M2 M*φ*. **(C)** Number of significantly up- and downregulated lncRNAs in M2 M*φ* compared to monocytes. LncRNAs with fold-change ≥2 were considered differentially expressed. **(D)** Heat map based elucidation of lncRNAs showing differential expression pattern in monocytes and M2 M*φ*. The numbers on the top of the heat map show the monocytes isolated from three donors for M2 M*φ* conversion. **(E−H)** Time kinetics of GAS5 and IPW expression in M1 and M2 M*φ* with respect to monocytes using RT-qPCR. Data were normalized with *β*-actin as a housekeeping control. Each bar is representative of at least three different experiments and represents the mean ± standard deviation. Two-tailed t-test was used to evaluate statistical significance. *P < 0.05, **P < 0.01, ***P < 0.001.

To evaluate the differential expression of lncRNAs during macrophage differentiation, sorted CD14^+^ monocytes from human PBMCs were treated with M-CSF for 7 days. **Figure 1A** shows the schematic representation of lncRNA profiling workflow. We profiled eighty-eight different lncRNAs using a focused PCR array and detected 49 lncRNAs in monocytes and/or M*φ* (with a cut-off value Ct =>34). Interestingly, six lncRNAs were expressed either in M*φ* (CDKN2B-AS1, HOXA-AS2, and NAMA) or monocytes (MIAT, HOXA-AS3, and PTENP1), thereby strongly suggesting a plausible role of lncRNAs in coordinating macrophage differentiation events (**Figure 1B**). Compared to monocytes, twelve lncRNAs were upregulated and five were downregulated in M2 M*φ* (fold change ≥2) (**Figure 1C**). CDKN2B-AS1, GAS5, HOXA-AS2, IPW, MALAT1, OIP5-AS1, HOTAIRM1, RBM5-AS1, SNHG16, TUG1, NAMA, and ZFAS1 were upregulated, while MIAT, HOXA-AS3, PTENP1, RN7SK, and NEAT1 were downregulated in M2 M*φ* (**Figure 1C**). **Figure 1D** shows the heat map of the expression profiles of differentially expressed lncRNAs. The fold change and *P* values of the differentially expressed lncRNAs are listed in **Table 1**. It can be noted that some lncRNAs showed higher *P* value (>0.05) but were included in the list because they were detected in two out of three donors (either monocyte or macrophage) but were consistently absent in other cell types or showed Ct very low values. These results show a unique acquisition of lncRNA species or expression changes during monocyte to macrophage differentiation.

**Table 1:** List of differentially expressed lncRNA identified in monocyte to M2 macrophage differentiation.

LncRNA	Fold change	P value
CDKN2B-AS1	224.20	0.000022
HOXA-AS2	39.84	0.000402
NAMA	92.94	0.000604
GAS5	3.05	0.104348
IPW	9.13	0.055467
MALAT1	6.74	0.000171
OIP5-AS1	3.20	0.031073
HOTAIRM1	2.68	0.109557
RBM5-AS1	3.38	0.079366
SNHG16	6.62	0.044420
TUG1	5.10	0.000726
ZFAS1	4.90	0.007734
RN7SK	−4.01	0.000275
NEAT1	−4.42	0.038312
HOXA-AS3	−4146.42	0.120368
MIAT	−341.64	0.011996
PTENP1	−65523339.50	0.000029

Upregulated lncRNAs are highlighted in red and downregulated lncRNAs are highlighted in blue.

In the above observation, the expression of a subset of lncRNAs is differentiation responsive. We therefore monitored the expression kinetics of selected lncRNAs GAS5 and IPW in M1 and M2 macrophages at 18 h, day 3, day 5, and day 7 of differentiation. Interestingly, the expression of GAS5 exhibits a similar expression profile in differentiating M1 and M2 M*φ*. Its expression increased approximately twofolds on day 3 and maintained at comparable levels on days 5 and 7 (~threefolds) (**Figures 1E, F**). IPW expression shows markedly differential pattern in M1 and M2 M*φ*. In M2 M*φ*, IPW levels decrease gradually until day 5 (~90%) and then sharply increase on day 7 (~25%) (**Figure 1G**). In M1 M*φ*, expression of IPW increased steadily until day 3 (~threefolds), drastically decrease at day 5 (~60%) and maintained at similar levels at day 7 (**Figure 1G**). Expression of GAS5 and IPW in day 7 M2 M*φ* corroborates with PCR array. This data clearly shows that lncRNAs are responsive to the extracellular milieu and might be involved in regulating macrophage polarization status.

### LncRNAs Regulate M1 and M2 Macrophage Polarization

Next, we asked whether the differentially expressed lncRNA directly affects macrophage polarization. To answer this, we silenced RN7SK (highly expressed in monocytes), GAS5, IPW, and ZFAS1 in M2M*φ* by siRNA transfection on day 3 and examined the changes in M1 and M2 phenotype markers on day 7. Using siGLO Red, we consistently obtain ~90% transfection efficiency in macrophages as observed under florescence microscope or flow cytometry (**Supplementary Figures 1A, B**).

Transient transfection of siRNA targeting candidate lncRNA resulted in approximately 70, 80, 50, and 80% reduction in the expression of RN7SK, GAS5, IPW, and ZFAS1 as compared to control (**Supplementary Figure 1C**). To evaluate the impact of siRNA on cell death, we performed cell viability assay in cells transfected with siRNA and did not observe any significant changes (data not shown). siRNA transfected M2M*φ* were evaluated for the expression of M1/M2 specific surface markers by flow cytometry at day 7. Gating strategy for the flow cytometric analysis and the percentages of gated cell population are shown in **Supplementary Figure 2A**. Single cells between FSC-Area and FSC-Height were included inside the slanted shape gate to examine the expression changes in M1 and M2 surface markers.

**Figure 2:**
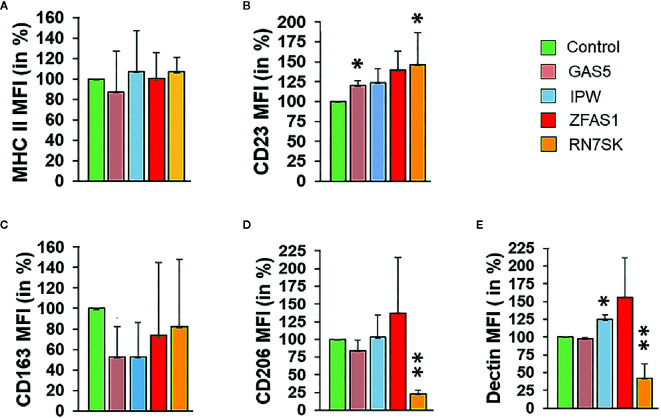
Knockdown of RN7SK and GAS5 skew macrophage polarization by suppressing M2 phenotype markers. Day 3 M2 macrophages were transfected with 100 nM siRNA targeting GAS5, IPW, ZFAS1, RN7SK or scramble control. After 72 h, the effect of lncRNA knockdown on the expression of **(A)** MHC II, **(B)** CD23 (surface markers for M1 M*φ*), **(C)** CD163, **(D)** CD206 and **(E)** Dectin (surface markers for M2 M*φ*) was assessed by flow cytometry. Histograms showing percent geometric mean fluorescence intensity (Geo. MFI) for five different polarization markers after lncRNA knockdown. Data are mean ± SEM of three independent experiments (n = 3). Two-tailed t-test was used to evaluate the statistical significance compared to control siRNA. *P < 0.05, **P < 0.01.

Interestingly, RN7SK knockdown resulted in significant upregulation of M1 markers, MHCII (~10%; *P* > 0.05) and CD23 (~30%; *P* < 0.05) and downregulation of all three M2 surface markers, CD206 (~80%; *P* < 0.01), dectin (~60%; *P* < 0.01), and CD163 (~18%; *P* > 0.05) levels (**Figures 2A–E**), and as compared to control (**Figure 2A**). Scatter dot plots show overall changes in the viable cell population for M1 and M2 markers (**Supplementary Figure 2B**). Knockdown of GAS5 (another differentially expressed lncRNA) significantly increased the levels of M1 marker MHCII (~20%; *P* < 0.05) (**Figures 2A–E**). Conversely, M2 markers CD163 (~45%; *P* < 0.05) and CD206 (~20%; *P* > 0.05) exhibited reduced expression in GAS5 knockdown, but the changes were not significant compared with control (**Figure 2**). CD23 (M1 marker) and Dectin (M2 marker) did not show any significant change in the expression in GAS5 knockdown. IPW knockdown induced the expression of M2 marker Dectin (20%; *P* < 0.05), whereas CD163 was downregulated (50%; *P* < 0.05). CD206 expression showed a similar expression compared to the control. On the other hand, IPW knockdown did not exhibit significant changes in the surface expression of M1 markers MHCII and CD23 (**Figures 2A–E**). Thus, the role of IPW in M*φ* polarization is not clear. This data substantiated the role of lncRNA in the M1/M2 switch. The effect of RN7SK can be considered as stronger as compared to IPW and GAS5. Taken together, our results indicate that lncRNA RN7SK promotes M2 phenotype.

Next, we examined whether lncRNA-mediated impact on the protein levels of M1/M2 markers occurs at the transcriptional levels. Expression levels of CD206, CD163, and CD23 transcripts were examined in M2 M*φ* transfected with siRNA targeting RN7SK and GAS5, which showed most significant changes in flow cytometric analysis. Compared to control siRNA, no significant changes in the expression of CD206, CD163, or CD23 were observed in GAS5 knockdown cells (**Supplementary Figures 3A–C**). However, knockdown of RN7SK, showed slight upregulation in the expression of CD163, albeit not significant compared to control or GAS5 (**Supplementary Figures 3A–C**). These results indicate that RN7SK and GAS5-mediated changes likely occur at the post-transcriptional or post-translation levels.

### RN7SK and GAS5 Augments Antigen Processing and Presentation

Macrophages are key antigen-presenting cells (APCs) that bridge innate and adaptive immunity (1–4). These cells recognize a diverse array of antigens, internalize them by endocytosis/phagocytosis, and subsequently present specific epitopes of processed antigens to T cells for efficient priming *via* MHC class I or II. We therefore examined if lncRNAs regulate antigen uptake and processing by M*Φ*. Cells were transfected with siRNA targeting GAS5, IPW, ZFAS1, and RN7SK and antigen uptake and processing were concurrently assessed by incubating with ovalbumin conjugated with Texas Red (for uptake) and BODIPY FL dye (DQ) ovalbumin (for processing). To assay the antigen uptake and processing, we harvested cells and quantitated fluorescence by flow cytometry.

**Figure 3:**
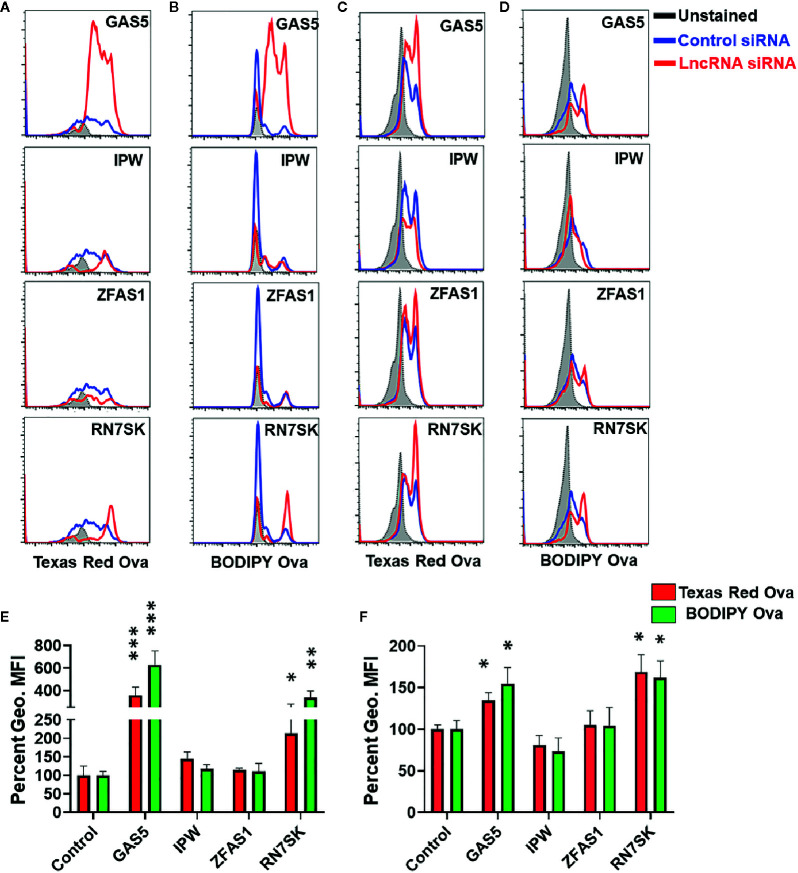
RN7SK and GAS5 silencing enhance antigen uptake and processing in macrophages. Day 3 M1 or M2 macrophages were transfected with 100 nM siRNA targeting GAS5, IPW, ZFAS1, RN7SK or scramble and their impact on antigen uptake and processing was analyzed after 72 h. Antigen uptake and processing were monitored by measuring Texas Red and BODIPY fluorescence, respectively. **(A–D)** Overlay of cell population histograms in control or lncRNA targeting siRNA to assess the differences in antigen uptake and processing. **(E–F)** Differential Mean fluorescence intensity (MFI) of Texas Red and BODIPY in M1 and M2 macrophages (lncRNA siRNA *vs* control siRNA). The ratio of geometric mean fluorescence intensity (geo. MFI) value of control and lncRNA targeting siRNA was obtained and were converted to percentages. Each bar is representative of at least three different experiments and represents the mean ± standard deviation. Two-tailed t-test was used to evaluate statistical significance. *P < 0.05, **P < 0.01, ***P < 0.001.

Overlay of histograms of control and lncRNA targeting siRNA show marked differences in antigen uptake and processing by lncRNAs (**Figures 3A, B**). Control siRNA normalized percent changes in lncRNA silenced M1 and M2 M*Φ* are shown for uptake and processing (**Figures 3C, D**). The antigen uptake and processing for three independent experiments are shown as percentage mean fluorescent intensity (MFI) for M1 and M2 macrophages, respectively (**Figures 3E, F**). Compared to control, knockdown of GAS5 and RN7SK augmented antigen uptake and processing in both M1 and M2 M*Φ* as observed by higher Texas Red and BODIPY signals, while a modest reduction in antigen uptake or processing was observed in IPW knockdown in M2 M*Φ* but not in M1 M*Φ* (**Figures 3A–D**). No significant differences were observed in ZFAS1 silenced cells. GAS5 knockdown induced Ova uptake and processing by ~3.5- and 6-folds, respectively, in M1 MΦ. Similarly, knockdown of GAS5 in M2 M*Φ* induced Ova uptake and processing by ~1.3- and ~1.5-folds, respectively (**Figures 3C, D**). In RN7SK silenced M1 M*Φ*, approximately 2- and 3.5-fold increase in antigen uptake and processing was observed, while knockdown of RN7SK in M2 M*Φ* enhanced antigen uptake and processing by ~1.5- and ~1.6-folds (**Figures 3C, D**). The knockdown of IPW in M2 M*Φ* showed a reduction in antigen uptake and processing by ~20%, albeit not significant. However, M1 M*Φ* showed an opposing effect of IPW silencing than M2 M*Φ*, with a slight increase in antigen uptake (~40%) and processing IPW (~15%) (**Figures 3C, D**).

### RN7SK and GAS5 Regulate Phagocytosis in Macrophages

**Figure 4:**
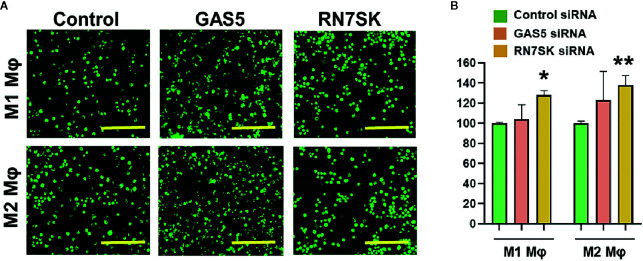
Knockdown of RN7SK augments bacterial phagocytosis in M1 and M2 macrophages. Day 3 M1 or M2 macrophages were transfected with 100 nM siRNA targeting GAS5, RN7SK or scramble. Phagocytosis was assayed using GFP labeled *E. coli* for 2 h by imaging and flow cytometry. **(A)** Representative images showing phagocytosed bacteria (green) in M1 and M2 macrophages transfected with lncRNA or control siRNA. Images were captured using fluorescent microscope. Scale bar—200 µm. **(B)** Cells harvested after phagocytosis assay and quantitative differences in phagocytic efficiency were measured. Geometric Mean Fluorescent intensity (Geo. MFI) of phagocytosed green *E. coli* in M1 and M2 macrophages was calculated for GAS5, RN7SK or control siRNA and percent changes were calculated with respect to control. Each bar is representative of at least three different experiments and represents the mean ± standard deviation. Two-tailed t-test was used to evaluate statistical significance (*P < 0.05, **P < 0.01).

Myeloid cells, including macrophages, phagocytose the invading pathogens, process and present antigenic epitopes to T cells that elicit efficient adaptive immune responses. After phagocytosis, these APCs migrate from tissues to lymph nodes and subsequently activate naive T cells (1–9). Both processes allow crosstalk of innate and adaptive immune cells. We examined the impact of RN7SK and GAS5, which regulated macrophage polarization and antigen uptake/processing, on phagocytosis by M1 and M2 polarized M*φ*. Cells were transfected with siRNA targeting RN7SK, GAS5, or control siRNA. After 72 h post-transfection, sufficient time for siRNA-mediated knockdown of target RNA, phagocytosis assays were performed using pHrodo™ Green labeled *E. coli*. In both M1 and M2 M*φ*, knockdown of RN7SK augmented phagocytosis but no significant impact was observed for GAS5. **Figure 4A** showed representative images of M1 and M2 M*φ* in control, RN7SK or GAS5 knockdown. Compared to control, higher intensity of green *E. coli* was observed in RN7SK knockdown cells. Flow cytometry analysis of harvested cells further confirmed this observation. Histograms show higher mean geometric fluorescent intensity (MFI) in RN7SK knockdown compared with control (**Figure 4B**). On the contrary, the GAS5 knockdown did not significantly affect bacterial uptake in M1 or M2 M*φ* (**Figures 4A, B**). Dot plots show a clear shift in the population towards right in RN7SK and GAS5 knockdown cells, indicating a higher uptake of *E. coli* by M1 and M2 M*φ* transfected with RN7SK or GAS5 siRNA compared to control (**Supplementary Figure 4**). Overall, these results indicate that differentially expressed lncRNAs regulate critical biological functions and polarization of macrophages.

## Discussion

Timely polarization, differentiation, and activation of macrophages are key to orchestrate the sequence of events leading to the immune response against invading pathogen. To date, multiple reports have consensus that concerted actions of pro- and anti-inflammatory cytokine milieu during pathogen proliferation drives M1 and M2 polarization of macrophages, respectively. Assessment of cytokine milieu can be misleading towards the assessment of macrophage dynamics due to unexpectedly high systemic presence of anti-inflammatory cytokines (IL-4, IL-6, IL-10, IL-1R*α*) during initial phases of certain infections, the short half-life of cytokine, and certain infection location in tissue may not be conducive for the collection of regional samples (31, 32). Therefore, there is a demand for sensitive and robust molecular markers to assess M1 and M2 macrophages that will advance our understanding of unique lncRNAs with M1/M2 specific expression.

LncRNAs exhibit cell-specific expression (11, 12, 18) and regulate epigenetic (histone modification and DNA methylation), post-transcription (RNA processing and mRNA stability) and translation pathways to govern multiple aspects of cellular functions (33–38). Therefore, in this study, we have profiled lncRNAs (using lncRNA PCR array) in sorted CD14^+^ monocytes and *in vitro* differentiated M2*φ* (*via* M-CSF treatment) in the absence of any pro- and anti-inflammatory cytokine. Of the 49 detected lncRNAs, three expressed exclusively in monocytes and three lncRNAs showed expression in M2 macrophages only. Among the up- and downregulated lncRNAs mentioned in **Table 1**, we selected GAS5, IPW, ZFAS1, and RN7SK to study their involvement in monocyte-to-macrophage polarization and innate immune functions. Our results showed that the lncRNAs**–**RN7SK and GAS5 influence macrophage polarization, antigen uptake/processing and phagocytosis.

Small nuclear RNA 7SK (RN7SK)—approximately 330 nts long and ubiquitously expressed noncoding RNA—is transcribed by RNA pol III (39–42). RN7SK negatively regulates RNA Polymerase II transcription elongation *via* binding to the positive transcription elongation factor b (P-TEFb). Interestingly, its interaction with HMGA1 high mobility group AT-hook competes with its binding to DNA (43, 44). By virtue of its regulatory role in transcription factor activity, RN7SK controls cellular differentiation, cell proliferation and senescence. RN7SK expression is significantly downregulated in stem cells and human tumor tissues, and its overexpression enhances (45–47). However, the role of RN7SK in macrophage polarization is not studied. RN7SK was identified as one of the most significantly altered lncRNA in our expression profiling. Interestingly, knockdown of RN7SK promotes M1 and suppresses M2 phenotype. Of the tested markers, M2-associated CD206 and dectin were downregulated in RN7SK knockdown, while M1 markers MHCII (not significant) and CD23 (significant) exhibit upregulation. However, we did not observe any significant changes in the expression of CD206, CD163, or CD23 mRNA in RN7SK knockdown cells. This observation suggests that RN7SK directly interacts with and alters the stability of aforementioned proteins at post-translation levels or may act through indirect regulatory factors that drive stability of surface proteins. LncRNAs act as modular regulatory RNA and their role in post-translational and post-transcriptional regulation is shown for different proteins and transcripts (11, 12, 22–24). Nonetheless, these observations strongly support that RN7SK favors the M1 phenotype by suppressing M2 markers at the protein levels. Augmented phagocytosis (of *E. coli*) and antigen uptake/processing further corroborated this observation. M1M*φ* are more potent in phagocytosis and antigen uptake/processing compared with M2M*φ*. Consistent with the surface marker expression analysis, we have also noticed that RN7SK knockdown in M2M*φ* augments phagocytosis of *E. coli*. Our results, for the first time, describe the role of RN7SK in skewing macrophage polarization and its regulation of key innate immune function in macrophages.

GAS5 locus generates ~4 kb long transcript, which yields 10 different snoRNAs (SNORD) and 24 mature RNA isoforms. GAS5 was identified in a screening of genes expressed in G0 serum-starved NIH/3T3 cells (48). Overexpression of GAS5 appeared as a strategy for the tumor therapeutics *via* inducing apoptosis and concomitant attenuated cell proliferation (49). Similar to the lncRNA RN7SK function, GAS5 also significantly increases MHCII (M1 marker) and inhibits CD163 and CD206 (albeit not significant) expression at the same time. At the RNA levels, GAS5 knockdown does not alter expression of CD206, CD163 or CD23 transcripts. In this context, it can be inferred that the GAS5 knockdown favors M1 but less strongly as compared to RN7SK. GAS5 is known to silence CCL1, a chemokine secreted by M2 macrophages and is shown to regulate macrophage polarization. Our results show that GAS5 is highly expressed in M1 macrophages compared to M2, and this expression increases with differentiation (compare Day 1 to Day 5 or 7) and corroborates with the previous studies (50, 51). However, unlike our findings of the pro-M1 phenotype by GAS5, other studies have shown a pro-M2 phenotype. Hu et al. showed that overexpression of GAS5 promoted macrophage (RAW) polarization toward an M1 phenotype by inducing nitric oxide synthase (iNOS), IL-1β, and TNF-α compared with the empty vector control (49). GAS5-mediated increase in the proinflammatory cytokines IL-6, IL-1β, and TNF-α was shown in ox-LDL-induced THP-1 macrophages (50). Mechanistically, GAS5 inhibits transcription Topoisomerase-Related Function 4 (TRF4), a key factor controlling M2 macrophage polarization (51). This discrepancy could be attributed to the differences in the experimental design, use of cell lines instead of primary cells, polarization marker analysis and macrophage differentiation. Our experiments were performed by transfecting lncRNA targeting siRNA at day 3 of M-CSF stimulated M2 macrophages and the surface expression of M1 and M2 markers is assessed at day 7. This allowed us to examine changes during the differentiation process and not in the polarized macrophages examined in the aforementioned studies.

## Conclusion

In summary, this study unravels the following imperative features—1) remarkable changes in lncRNA profile are associated with monocyte-to-macrophage differentiation, 2) knockdown of RN7SK and GAS5 downregulates M2 markers and upregulates M1 markers suggesting that lncRNAs regulate macrophage polarization by skewing them towards M2 phenotype, and 3) RN7SK negatively regulates antigen uptake/processing and bacterial phagocytosis. Overall, our findings describe a key role of lncRNAs in macrophage differentiation, polarization, and regulating innate functions including antigen processing and phagocytosis.

## Data Availability Statement

The raw data supporting the conclusions of this article will be made available by the authors, without undue reservation.

## Author Contributions

AN conceived the study. AN, IA, and AV performed the experiments. AN, IA, AV, and RN analyzed the data. AN and RN wrote the manuscript. All authors contributed to the article and approved the submitted version.

## Funding

This work was funded by the NIH/NIDCR R03 DE027147, R01DE027980, and R21DE026259 to AN.

## Conflict of Interest

The authors declare that the research was conducted in the absence of any commercial or financial relationships that could be construed as a potential conflict of interest.

## Supplementary Material

Supplementary Figure 1 Efficient siRNA-mediated silencing of lncRNA in macrophages. Fluorescent oligos (siGLO Red) were used as positive control for transfection. **(A)** Image showing uptake of siGLO Red confirming the successful transfection. **(B)** Flow cytometric analysis of siGLO Red positive (~90%) M2 macrophages. **(C)** Macrophages were transfected with 100 nM siRNA targeting GAS5, IPW, ZFAS1, RN7SK or control and the cells were harvested after 72 h. The expression of lncRNAs was quantified by RT-qPCR and the fold change was calculated with respect to control siRNA. GAPDH was used as a housekeeping control. Each bar is representative of at least three different experiments and represents the mean ± standard deviation. Two-tailed t-test was used to evaluate statistical significance. ***P < 0.01, ***P < 0.001*.

Supplementary Figure 2 Gating strategy and cell population used for M1 and M2 surface marker expression by flow cytometry. **(A)** Schematic view of the gating strategy used for flow cytometric analysis. Scatter dot plots showing the expression of **(B)** M1 and **(C)** M2 surface markers.

Supplementary Figure 3 Expression of M1 and M2 marker transcripts is not impacted by lncRNA knockdown. M1 or M2 macrophages were transfected with 100 nM siRNA targeting GAS5, RN7SK or control and total RNA was isolated after 72 h. Expression of **(A)** CD206, **(B)** CD163 and **(C)** CD23 was quantified by RT-qPCR and the fold change was calculated with respect to control siRNA. GAPDH was used as a housekeeping control. Each bar is representative of at least three different experiments and represents the mean ± standard deviation. Two-tailed t-test was used to evaluate statistical significance.

Supplementary Figure 4 Scatter dot plots showing cell population of M1 and M2 macrophages after phagocytosis assay. **(A)** Schematic view of the gating strategy used for phagocytosis assay analysis. Dot plots of population showing the phagocytosis of pHrodo Green labelled *E. coli* by **(B)** M1 and **(C)** M2 macrophages.
