# Supplementary figures and images for: Long Non-coding RNAs RN7SK and GAS5 Regulate Macrophage Polarization and Innate Immune Responses
Source: Front Immunol. 2020 Dec 9;11:604981. doi: 10.3389/fimmu.2020.604981 (PMC7757381; doi:10.3389/fimmu.2020.604981)

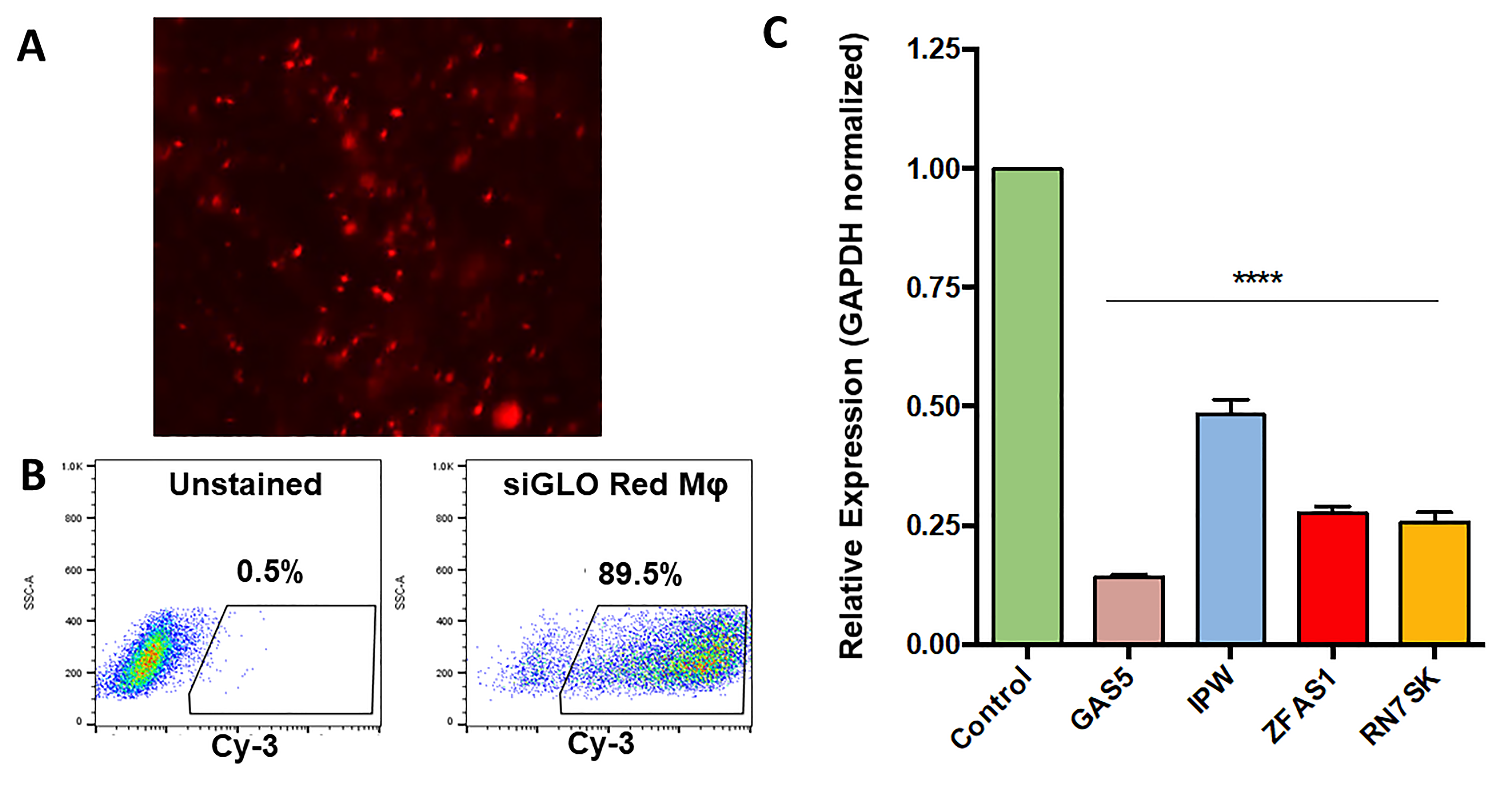

Supplement: Supplementary Figure 1 — Efficient siRNA-mediated silencing of lncRNA in macrophages. Fluorescent oligos (siGLO Red) were used as positive control for transfection. (A) Image showing uptake of siGLO Red confirming the successful transfection. (B) Flow cytometric analysis of siGLO Red positive (~90%) M2 macrophages. (C) Macrophages were transfected with 100 nM siRNA targeting GAS5, IPW, ZFAS1, RN7SK or control and the cells were harvested after 72 h. The expression of lncRNAs was quantified by RT-qPCR and the fold change was calculated with respect to control siRNA. GAPDH was used as a housekeeping control. Each bar is representative of at least three different experiments and represents the mean ± standard deviation. Two-tailed t-test was used to evaluate statistical significance. **P < 0.01, ***P < 0.001. [file Image_1.tif]

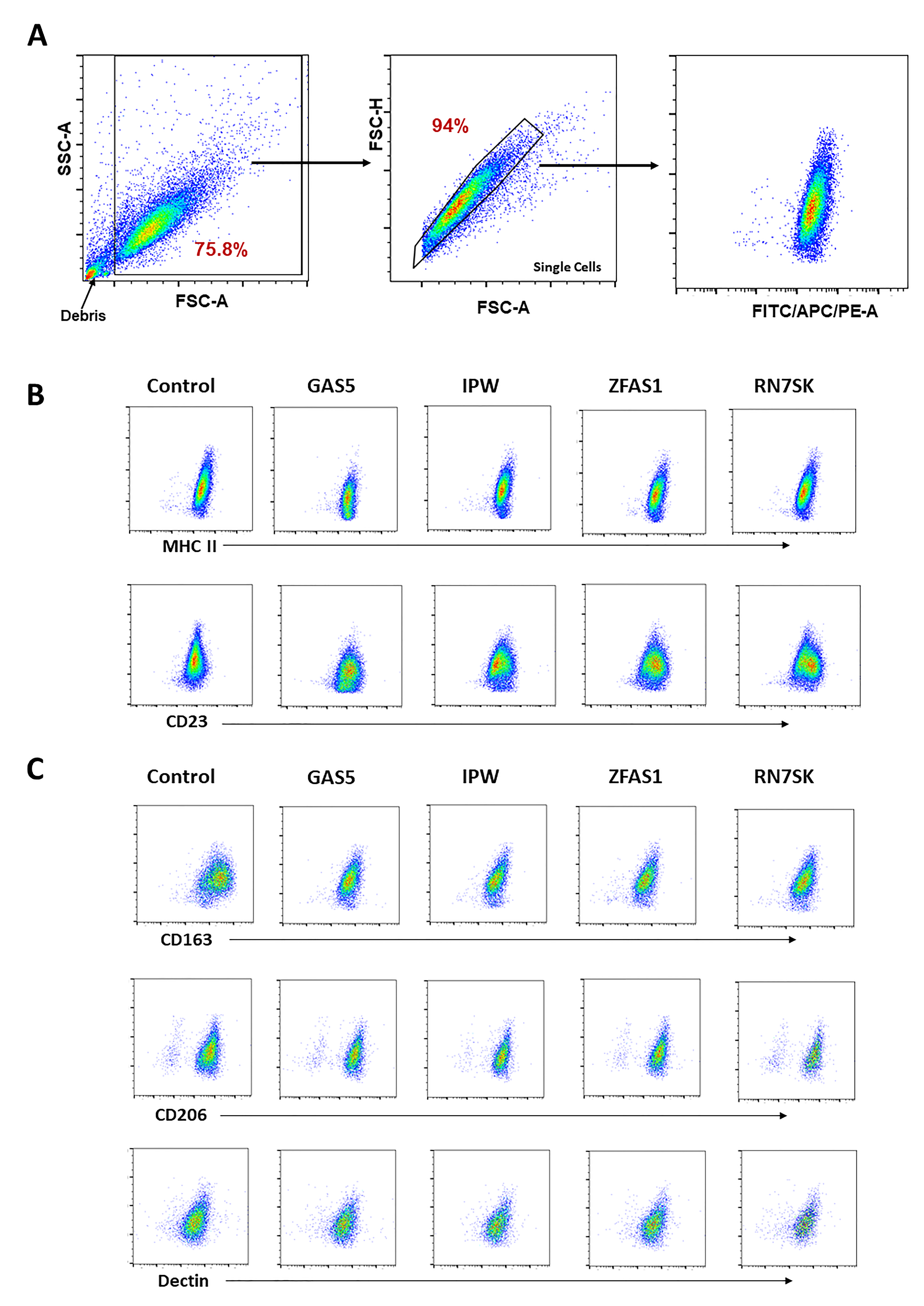

Supplement: Supplementary Figure 2 — Gating strategy and cell population used for M1 and M2 surface marker expression by flow cytometry. (A) Schematic view of the gating strategy used for flow cytometric analysis. Scatter dot plots showing the expression of (B) M1 and (C) M2 surface markers. [file Image_2.tif]

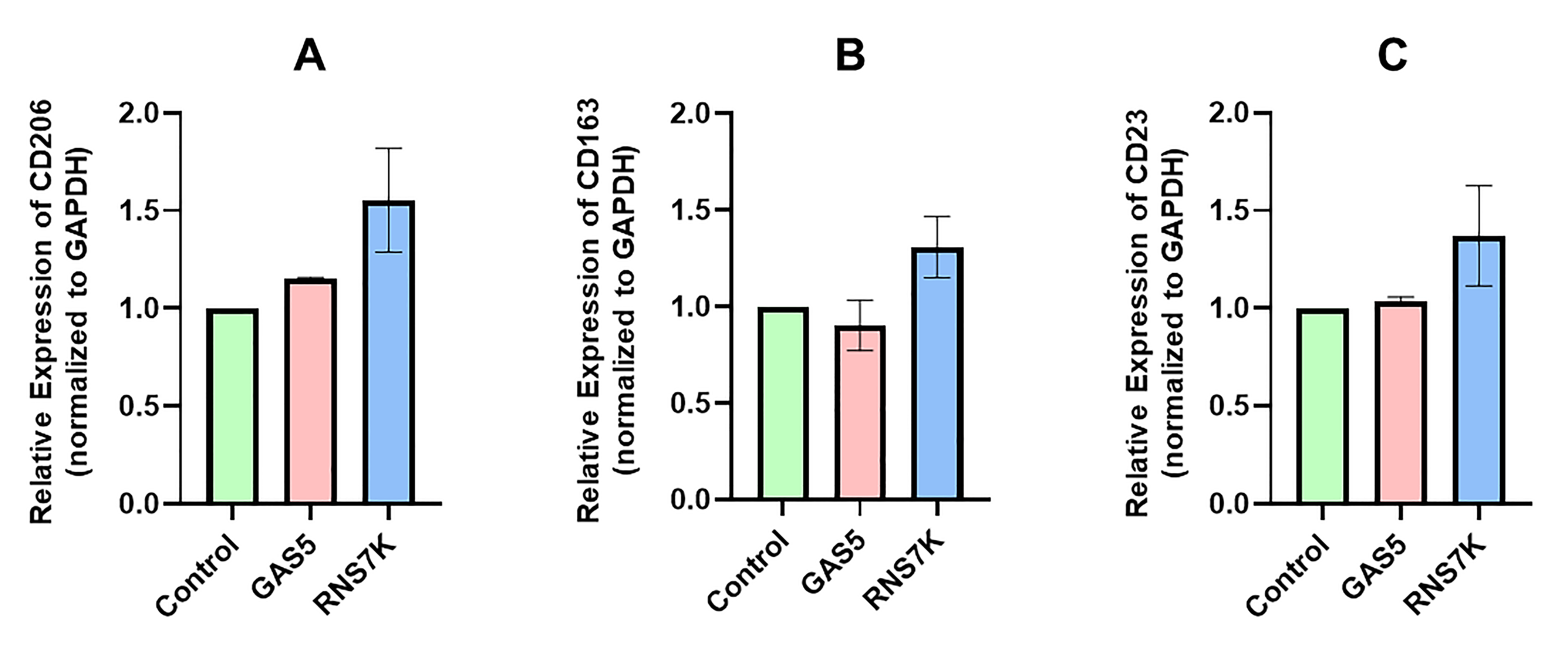

Supplement: Supplementary Figure 3 — Expression of M1 and M2 marker transcripts is not impacted by lncRNA knockdown. M1 or M2 macrophages were transfected with 100 nM siRNA targeting GAS5, RN7SK or control and total RNA was isolated after 72 h. Expression of (A) CD206, (B) CD163 and (C) CD23 was quantified by RT-qPCR and the fold change was calculated with respect to control siRNA. GAPDH was used as a housekeeping control. Each bar is representative of at least three different experiments and represents the mean ± standard deviation. Two-tailed t-test was used to evaluate statistical significance. [file Image_3.tif]

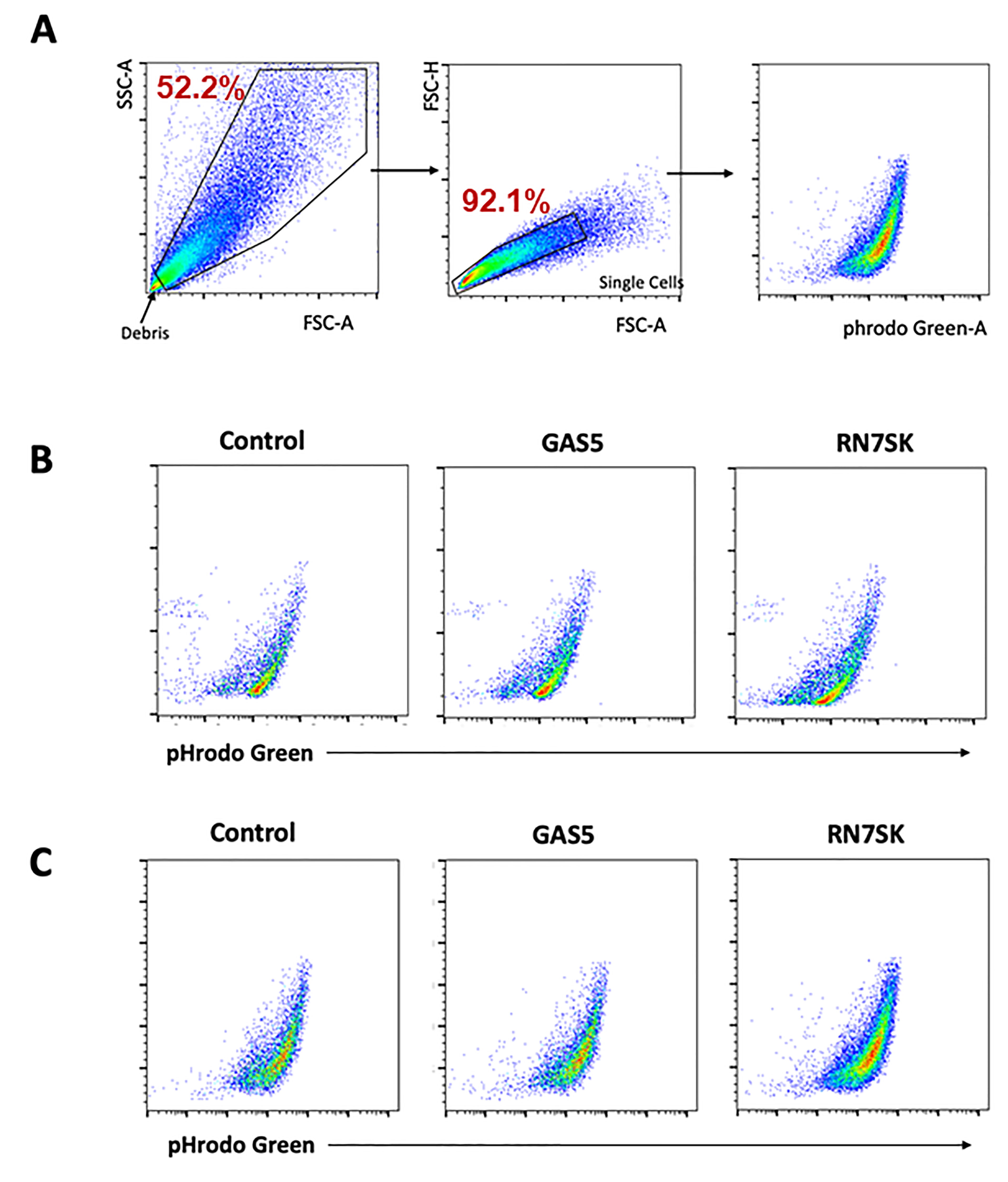

Supplement: Supplementary Figure 4 — Scatter dot plots showing cell population of M1 and M2 macrophages after phagocytosis assay. (A) Schematic view of the gating strategy used for phagocytosis assay analysis. Dot plots of population showing the phagocytosis of pHrodo Green labelled E. coli by (B) M1 and (C) M2 macrophages. [file Image_4.tif]
